# Genomic and phenotypic profiling of an *Artemisia* endophyte: dual biostimulant and biocontrol activities in pea under *Fusarium* stress

**DOI:** 10.3389/fmicb.2025.1643204

**Published:** 2025-08-29

**Authors:** Shervin Hadian, Donald L. Smith, Skaidre Suproniene

**Affiliations:** ^1^Microbiology Laboratory, Institute of Agriculture, Lithuanian Research Centre for Agriculture and Forestry, Kėdainiai, Lithuania; ^2^Department of Plant Science, McGill University, Montreal, QC, Canada

**Keywords:** *Artemisia*, biocontrol, biostimulants, endophytic bacteria, *Serratia*

## Abstract

**Objective:**

To evaluate the plant growth-promoting and disease-suppressing potential of *Serratia* sp. AR11, an endophytic bacterium isolated from *Artemisia absinthium*, through phenotypic assessment and whole-genome analysis in pea (*Pisum sativum*) under normal and *Fusarium*-stress conditions.

**Materials and methods:**

Greenhouse experiments were conducted to assess the effects of AR11 inoculation on shoot and root growth, biomass, chlorophyll content, and *Fusarium*-induced stunting. Whole-genome sequencing was performed using the PacBio SMRT platform, followed by functional annotation to identify genes related to nutrient metabolism, secondary metabolite biosynthesis, and stress adaptation. Biosafety assessment included screening for virulence and antibiotic resistance genes.

**Results:**

AR11 inoculation significantly increased shoot and root length and biomass, while reducing *Fusarium*-induced stunting by over 70%. Under pathogen stress, treated plants showed a 67% increase in SPAD index compared to controls. Genome analysis revealed a complete 5.49 Mb circular genome with 5,175 protein-coding genes, including those for nitrogen metabolism, phosphate solubilization, siderophore production, and antifungal secondary metabolite biosynthesis. Additional features included stress-responsive regulatory systems, CRISPR-Cas loci, and DNA methylation patterns. No high-risk virulence or acquired antibiotic resistance genes were detected.

**Conclusion:**

*Serratia* sp. AR11 is a safe, multifunctional endophytic bacterium with strong genomic and phenotypic evidence for enhancing pea growth and suppressing *Fusarium*-related diseases, making it a promising candidate for sustainable low-input agriculture.

## Introduction

Promoting sustainable agriculture requires innovative solutions that minimize chemical inputs while enhancing crop productivity and resilience (Sudheer et al., 2020). Endophytic bacteria are a key part of the plant microbiome. Most live inside plant tissues without causing harm. These bacteria can improve plant growth, increase stress tolerance, and help protect against disease (Anand et al., 2023). These bacteria can fix atmospheric nitrogen, solubilize phosphate, produce plant hormones, and synthesize antimicrobial metabolites traits essential for biostimulant and bioprotective applications (Timofeeva et al., 2023). However, identifying highly effective strains and understanding the molecular mechanisms behind their beneficial effects remain major challenges.

Medicinal plants are an underexplored source of endophytic diversity with high biosynthetic potential (Aswani et al., 2020). Among them, *Artemisia* species have drawn attention to their rich secondary metabolite profiles and ethnobotanical importance (Lantzouraki et al., 2023). *Artemisia* species, such as *A. absinthium*, *A. alba*, *A. annua*, *A. verlotiorum*, and *A. vulgaris*, are recognized for their secondary metabolic. Caffeoyl and feruloyl quinic acid esters are identified as their potential bioactive compounds, which notably characterize the most antioxidant-rich species (Negri et al., 2024). Host plants and their endophytes often develop specialized relationships, a result of long-term co-evolution. These interactions can influence both the plant’s metabolic profile and the functional traits of the endophytes (Jia et al., 2016). The chemically rich environment of medicinal plants such as *Artemisia* may shape endophyte communities capable of producing diverse secondary metabolites (Tripathi et al., 2025). In some cases, the therapeutic properties of the plant may result, at least in part from metabolites synthesized by its endophytes. For example, *Artemisia nilagirica* and *A. absenithium* host endophytic bacteria known to produce antibacterial compounds (Ashitha et al., 2019; Damavandi et al., 2023). Endophytes, particularly those found in medicinal plants with ethnobotanical significance, act as promising sources of novel bioactive compounds (Nongkhlaw and Joshi, 2015). They produce a range of compounds that support plant growth and enhance protection against pathogens (Basit et al., 2021). Endophytes from *Artemisia* spp. not only contribute to host metabolism but may also enhance plant health in other crops (Hadian et al., 2024). Despite increasing recognition of their bioactive potential, few studies have investigated the functional genomics of *Artemisia* derived endophytes, particularly in relation to agriculturally important crops like pea (*Pisum sativum* L.).

Pea is a globally important legume that contributes to soil fertility through biological nitrogen fixation (Dhillon et al., 2022). However, it is highly susceptible to root rot caused by *Fusarium* spp., leading to yield losses exceeding 90% in severe cases (El-Saadony et al., 2021). While chemical fungicides remain a common control strategy, they pose environmental risks and are often ineffective under field variability. Harnessing endophytic microbes as dual function bioinoculants enhances growth while suppressing pathogens represents a promising but underdeveloped strategy in legume systems.

Endophytic microorganisms can suppress invading pathogens through multiple mechanisms, including competition for nutrients, and ecological niches within the rhizosphere, rhizoplane, and internal plant tissues. Certain beneficial endophytic bacteria produce antibiotics and lytic enzymes that actively inhibit pathogen growth. Additionally, introducing endophytic microbes into plants through artificial inoculation can significantly reduce infections caused by fungi, bacteria, viruses, insects, and nematodes, serving as an effective biological control strategy (Fontana et al., 2021; Hallmann et al., 1997). Numerous studies highlight the potential of endophytic bacteria in promoting plant growth and protecting against *Fusarium* infections. For example, it has been reported that *Pseudomonas aeruginosa* and *Aneurinibacillus aneurinilyticus* isolated from *Ocimum sanctum* significantly enhances pea growth and provides strong protection against *Fusarium oxysporum* f.sp. *pisi*, a major root rot pathogen (Gupta et al., 2022).

In our previous work, we isolated 61 endophytic bacterial strains from the roots, stems, and leaves of four *Artemisia* species (*A. absinthium*, *A. campestris*, *A. dubia*, and *A. vulgaris*) collected across diverse agroecological zones. Initial *in vitro* screening for key plant growth-promoting traits, such as phosphate solubilization, nitrogen fixation, and seed germination enhancement in pea (*Pisum sativum*) led to the selection of seven promising strains for further study (Hadian et al., 2024). Among these, strains AR11 and AR32, initially classified as *Bacillus* spp. via 16S rRNA gene sequencing, showed potential ecological relevance by modulating microbial diversity in the pea rhizosphere (Hadian et al., 2025). Despite growing recognition of the potential of endophytic bacteria in sustainable agriculture, the ecological functions and genomic characteristics of *Artemisia*-associated endophytes remain largely underexplored, particularly within legume cropping systems. While traits such as nitrogen fixation and phosphate solubilization have been individually reported, integrated studies that link genomic insights with plant performance under biotic stress are still limited. This is especially relevant for managing *Fusarium* wilt, a major challenge in pea (*Pisum sativum*) cultivation worldwide.

While many studies stop at phenotypic evaluation, our research integrates functional screening with whole-genome analysis to determine the molecular basis of beneficial traits. This phenotypic-to-genomic approach remains underexplored for endophytes from medicinal plants like *Artemisia*, particularly in pea systems. To address this, the present study combines phenotypic evaluation of seven *Artemisia*-derived endophytic bacterial strains with genome-based analysis under controlled greenhouse conditions, both with and without *Fusarium* inoculation. This approach enabled the identification of strains with consistent growth-promoting and disease-suppressing effects. The most effective strain was further investigated at the genomic level, revealing key traits related to plant growth support, stress response, and antifungal potential. The results provide valuable insights into the functional capacity of *Artemisia*-associated endophytes and support their potential use as bioinoculants in pea production.

We hypothesize that endophytic bacteria from *Artemisia* promote pea growth and reduce *Fusarium* infection through specific molecular traits. These include genes for nitrogen metabolism, phosphate solubilization, and antifungal compound production. By linking plant performance with genomic data, we aim to identify the key mechanisms behind this dual functionality.

## Materials and methods

### Isolation and selection of endophytic bacteria

Endophytic bacterial strains used in this study were previously isolated from root, stem, and leaf tissues of four *Artemisia* species (*A. absinthium*, *A. campestris*, *A. dubia*, and *A. vulgaris*), collected from three different regions in Lithuania: Kaunas, Kėdainiai, and Šiauliai. A total of 61 isolates were obtained, and seven strains were selected based on beneficial traits, including phosphate solubilization, nitrogen fixation, enhancement of pea seed germination, and antagonistic activity against *Fusarium* spp., as assessed by dual-culture assays. These results have been previously published (Hadian et al., 2024). The selected strains were: AR11, AR32, and AR35 (from *A. absinthium* root); VL13 (from *A. vulgaris* leaf); VR24 (from *A. vulgaris* root); CR25 (from *A. campestris* root); and DR31 (from *A. dubia* root).

### Assessment of plant growth-promoting activity and biocontrol potential in pea

#### Seed inoculation with endophytic bacteria

Pea seeds (*Pisum sativum* L.) were surface sterilized following a standard protocol. Sterilized seeds were immersed in bacterial suspensions (OD₆₀₀ = 1.0, approximately 10^8^ CFU mL^−1^) and incubated for 3 h at room temperature with gentle shaking to ensure uniform coating. Control seeds underwent the same procedure but were treated with sterile distilled water.

#### Soil preparation

Soil was mixed with commercial compost and sieved through a 2 mm mesh to remove debris. The final soil mix had an organic carbon content of 11.99%, total nitrogen of 1.084%, and a slightly alkaline pH of 7.79, which is favorable for nutrient availability and microbial activity. Macronutrient analysis showed 460 mg kg^−1^ of potassium oxide (K₂O) and 205 mg kg^−1^ of phosphorus pentoxide (P₂O₅), indicating high fertility suitable for plant growth and microbial interaction studies.

### Preparation of pathogenic inoculum

The pathogenic *Fusarium* sp. used in this study was previously isolated from infected pea roots and identified at the Microbiology Department of LAMMC (Hadian et al., 2024). Molecular analysis showed high similarity to *Fusarium oxysporum*, though species-level resolution was not the primary focus. Pathogenicity tests confirming its virulence on pea were thoroughly performed as described in Hadian et al. (2024), ensuring its suitability for the present experiments. Mycelial plugs (~5 mm) from PDA stock cultures were transferred to fresh potato dextrose agar (PDA) plates and incubated at 25 °C for 4 days. After 1 week of growth, conidia were harvested by flooding the plates with sterile distilled water and gently scraping the surface. The resulting suspension was filtered through sterile muslin cloth, and spore concentration was adjusted to 1 × 10^4^ conidia mL^−1^ using a hemocytometer. The inoculum was applied in two stages to ensure effective root-zone colonization: the first application was conducted at the first true leaf stage, and the second 1 week later by injecting 5 mL of the spore suspension into the soil near the root zone. Control plants received an equal volume of sterile distilled water (Raza et al., 2024).

### Experimental design and pot trials

The experiment was conducted under controlled greenhouse conditions (25 ± 2 °C, 16 h photoperiod, light intensity of ~250 μmol m^−2^ s^−1^, 60% relative humidity), where pea seeds were subjected to various treatments and sown in pots containing sterilized soil. The pots were maintained under optimal environmental conditions with regular irrigation to ensure adequate soil moisture. The experimental design included several treatment groups. The control group consisted of water-primed seeds without any bacterial or fungal inoculation. Another group received bacterial inoculation only, in which seeds were treated with one of the selected bacterial strains (AR11, AR32, AR35, VL13, VR24, CR25, and DR31) without exposure to *Fusarium*. A separate group, designated as *Fusarium* inoculation only (Control + Fu), included seeds that were challenged solely with the *Fusarium* pathogen, without bacterial treatment. The final group involved co-inoculation, where seeds were treated with a bacterial strain and simultaneously challenged with *Fusarium*. Each treatment was replicated 4 times, and the plants were observed for a period of 14 days following pathogen inoculation. Fourteen days after *Fusarium* inoculation, the plants were carefully uprooted and washed with tap water to remove soil residues. The root systems were then visually examined and scored for disease severity using a standardized 1–5 scale, where a score of 1 indicated healthy roots with no visible symptoms, 2 represented up to 25% root tissue damage, 3 indicated approximately 50% damage, 4 corresponded to around 75% root tissue damage, and 5 denoted complete damage or decay of root tissues.

### Measurement of plant growth parameters

Morphological parameters including root length, shoot length, fresh and dry weight of both roots and shoots were measured for each plant at the time of harvest. These values were used to assess the overall impact of treatments on plant growth and development.

### Chlorophyll content estimation

Chlorophyll content was determined using a SPAD-502 Plus chlorophyll meter (Konica Minolta, Japan). SPAD readings were taken from three fully expanded, healthy leaves, one each from the upper, middle, and lower canopy, to minimize variability. The meaning of the three readings was calculated to obtain the final SPAD value for each replicate.

### DNA extraction

Genomic DNA was extracted from the endophytic bacterial strain *Serratia* sp. AR11 using the GeneJET Genomic DNA Purification Kit (Thermo Scientific, United States) following the manufacturer’s protocol. Briefly, single colonies were inoculated into Luria-Bertani (LB) broth and incubated overnight at 30 °C with shaking at 200 rpm. Cells were harvested by centrifugation at 10,000 × g for 5 min. Lysis and protein precipitation steps were followed by ethanol-based DNA purification. The quantity and quality of the extracted genomic DNA were assessed using a Nanodrop ND-1000 spectrophotometer (Thermo Fisher Scientific, United States), and DNA was then submitted for high-throughput whole-genome sequencing.

### Whole-genome sequencing and bioinformatics analysis

High-molecular-weight genomic DNA from strain AR11 was submitted to Novogene (Germany) for long-read sequencing on the PacBio Sequel II platform using Circular Consensus Sequencing (CCS) mode to generate high-fidelity (HiFi) reads. Raw data underwent quality control and filtering prior to downstream analysis. *De novo* genome assembly was performed using Canu v1.5, optimized for long-read sequencing data. Circularization of contigs was completed using Circlator v1.5.5, and genome completeness was evaluated using BUSCO v5.4.3 with the bacterial single copy ortholog database. Structural genome annotation included gene prediction with Prodigal v2.6.3 (Hyatt et al., 2010), rRNA identification with Infernal v1.1.3 (Nawrocki and Eddy, 2013) using Rfam covariance models, and tRNA detection using tRNAscan-SE v2.0 (Chan and Lowe, 2019). Interspersed and tandem repetitive elements were annotated with RepeatMasker v4.0.5 (Tarailo-Graovac and Chen, 2009). Mobile genetic elements were identified using multiple tools: PhiSpy v2.3 (Akhter et al., 2012) for prophage prediction, IslandPath-DIMOB v0.2 (Bertelli and Brinkman, 2018) for genomic island detection, and CRT v1.2 (Bland et al., 2007) for CRISPR array identification. Secondary metabolite biosynthetic gene clusters (BGCs) were predicted with antiSMASH v5.0.0 (Blin et al., 2019). Functional annotation of predicted coding sequences was conducted using BLASTp (Altschul et al., 1990) (*E*-value ≤1 × 10^−5^) against the NCBI NR, Swiss-Prot, KEGG, and eggNOG databases. Gene Ontology (GO) terms were assigned via Blast2GO (Conesa et al., 2005), and functional classification into Clusters of Orthologous Groups (COGs) was performed using eggNOG-mapper. Carbohydrate-active enzymes (CAZymes) were identified using the CAZy (Cantarel et al., 2009) database, and membrane transporters were classified via the Transporter Classification Database (TCDB) (Saier, 2006). SignalP (Petersen et al., 2011) and TMHMM (Krogh et al., 2001) were used to predict signal peptides and transmembrane domains, respectively, to infer subcellular localization and secretion potential. Finally, DNA methylation profiling was carried out using the raw PacBio sequencing signal via SMRT Link v8.0.0, enabling genome-wide detection of modified bases including N6-methyladenine (m6A) and N4-methylcytosine (m4C), as well as associated methylation motifs and potential regulatory functions.

### Statistical and data analysis

All statistical analyses were conducted using R software (v4.2.3; https://cran.r-project.org/; accessed on 10 January 2024). Prior to hypothesis testing, data were evaluated for normality using the Shapiro–Wilk test and for homogeneity of variance using Levene’s test. For parametric variables (shoot height, root length, biomass, SPAD index), one-way analysis of variance (ANOVA) was performed, followed by Tukey’s honest significant difference (HSD) test for *post hoc* comparisons at a significance level of *p* < 0.05. For non-parametric data, such as disease severity scores, the Kruskal–Wallis test was applied, followed by Dunn’s post hoc test with Benjamini–Hochberg correction for multiple comparisons. To assess potential associations between physiological parameters, Pearson’s correlation analysis was performed, with significance set at *p* < 0.05. All values are reported as means ± standard error (SE). Each treatment group included four biological replicates (*n* = 4). Graphical visualizations were generated using the ggplot2 package in R.

## Results

### Impact of *Artemisia* derived endophytic bacteria on growth and biomass of pea plants under *Fusarium* stress

Seven bacterial strains were isolated from the endosphere of *Artemisia* spp. and selected based on previously demonstrated nitrogen fixation and phosphate solubilization activity (Hadian et al., 2024). Taxonomic analysis via 16S rRNA gene sequencing classified six isolates as *Bacillus* spp., while one strain (CR25) was identified as *Pseudomonas*.

### Growth promotion under non-stress conditions

**Figure 1:**
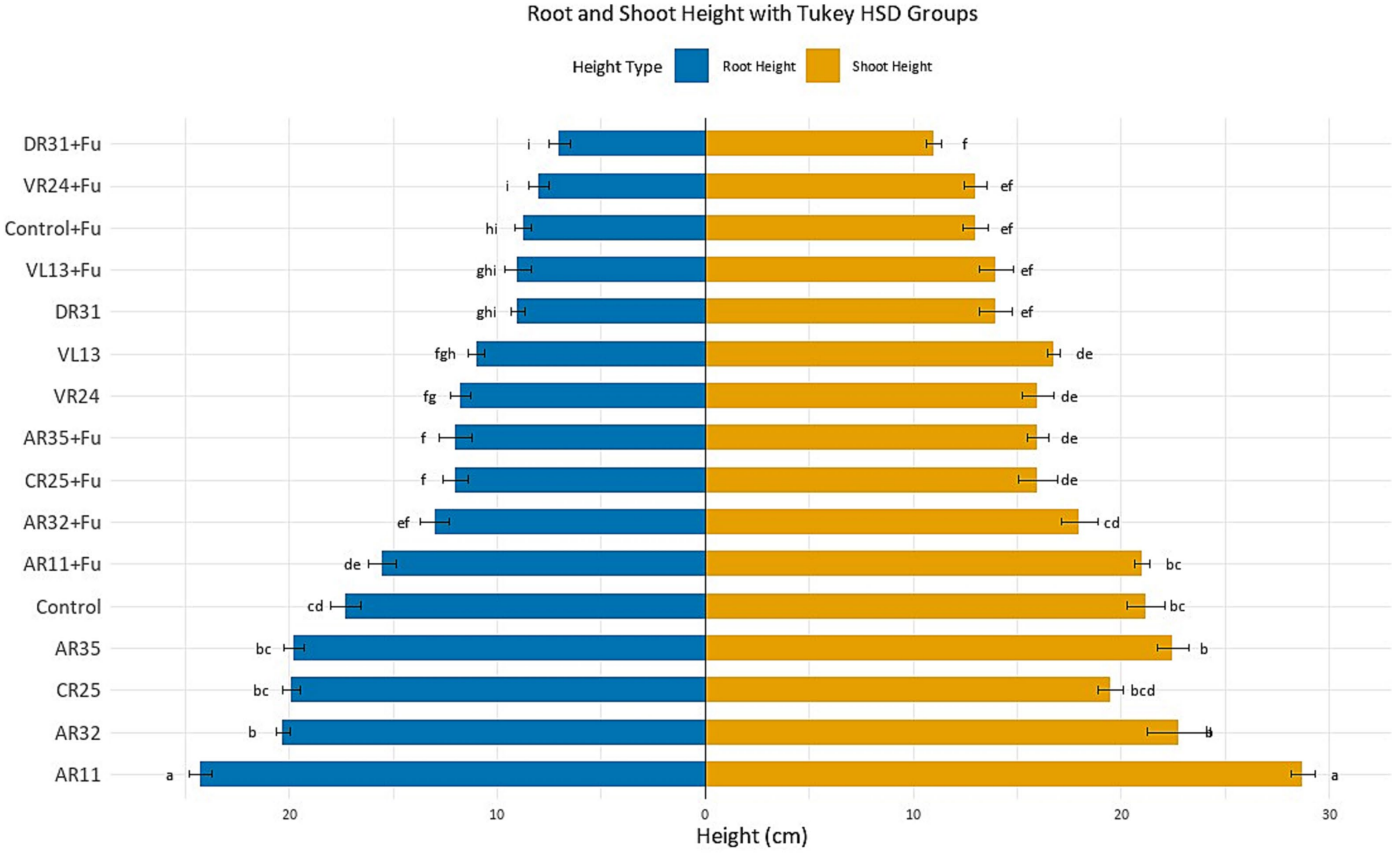
Effect of endophytic bacterial inoculation on pea root and shoot lengths under *Fusarium* stress. Bars show mean ± SE (*n* = 4). Significant differences (*p* < 0.05, one-way ANOVA, Tukey’s HSD). Different letters above bars indicate significant differences.

Under optimal (non-stress) conditions, inoculation with endophytic bacteria significantly enhanced pea plant growth compared to the uninoculated control (*p* < 0.05; Figure 1). Among all treatments, AR11 exhibited the most pronounced growth-promoting effect, with plants reaching 24.27 ± 0.5 cm root and 28.75 ± 0.7 cm shoot height. These values were assigned to the highest Tukey HSD groupings (“a”), confirming a statistically significant improvement (*p* < 0.05) over all other treatments. AR32 and AR35 also significantly increased root and shoot heights compared to the control (*p* < 0.05). Plants treated with AR32 reached 20.30 ± 0.6 cm root and 25.75 ± 0.6 cm shoot height, placing them in groups “b” and “b,” respectively. AR35 induced root and shoot heights of 19.75 ± 0.4 cm and 22.50 ± 0.6 cm, grouped as “bc” for both parameters. In contrast, control plants reached only 17.28 ± 0.4 cm root (group “cd”) and 22.30 ± 0.5 cm shoot (group “bc”), confirming a significant difference from AR11 and AR32 (*p* < 0.05). CR25 showed intermediate performance (19.40 ± 0.6 cm root, group “bc”; 21.90 ± 0.6 cm shoot, group “bcd”), which was not significantly different from AR35 but still improved over the control in root height (*p* < 0.05). In contrast, the remaining strains (VR24, VL13, and DR31) demonstrated only moderate or limited growth promotion. VR24-inoculated plants exhibited 15.05 ± 0.6 cm root (group “fg”) and 18.50 ± 0.6 cm shoot (group “de”), both significantly lower than AR11, AR32, and AR35 (*p* < 0.05). VL13 showed similar performance (14.50 ± 0.5 cm root, group “fgh”; 18.15 ± 0.6 cm shoot, group “de”), while DR31 achieved 12.00 ± 0.4 cm root (group “ghi”) and 17.25 ± 0.4 cm shoot (group “ef”), placing it among the least effective strains.

### Growth performance under *Fusarium* stress

*Fusarium* infection significantly reduced plant growth, as evidenced by the non-inoculated infected control, which showed root and shoot lengths of 8.75 ± 0.3 cm and 13.00 ± 0.4 cm, respectively. These values fell into Tukey groups “hi” (root) and “ef” (shoot), confirming a strong negative effect of the pathogen on pea development (*p* < 0.05; Figure 1). In contrast, inoculation with AR11 significantly mitigated the effects of *Fusarium* stress, with treated plants reaching 15.50 ± 0.4 cm root height and 21.00 ± 0.5 cm shoot height, grouped as “de” and “cd,” respectively. These values were the highest among all *Fusarium*-inoculated treatments, indicating that AR11 provided the most effective protection against pathogen-induced growth suppression (*p* < 0.05). AR32 and AR35 also conferred notable, though comparatively less, protective effects. AR32-treated plants reached 13.00 ± 0.3 cm root (group “ef”) and 19.25 ± 0.5 cm shoot (group “de”), while AR35 recorded 12.00 ± 0.4 cm root (group “f”) and 18.75 ± 0.6 cm shoot (group “de”). Though both strains significantly improved growth compared to the infected control (*p* < 0.05), they remained statistically inferior to AR11, as confirmed by non-overlapping Tukey groupings.

### Biomass accumulation

**Figure 2:**
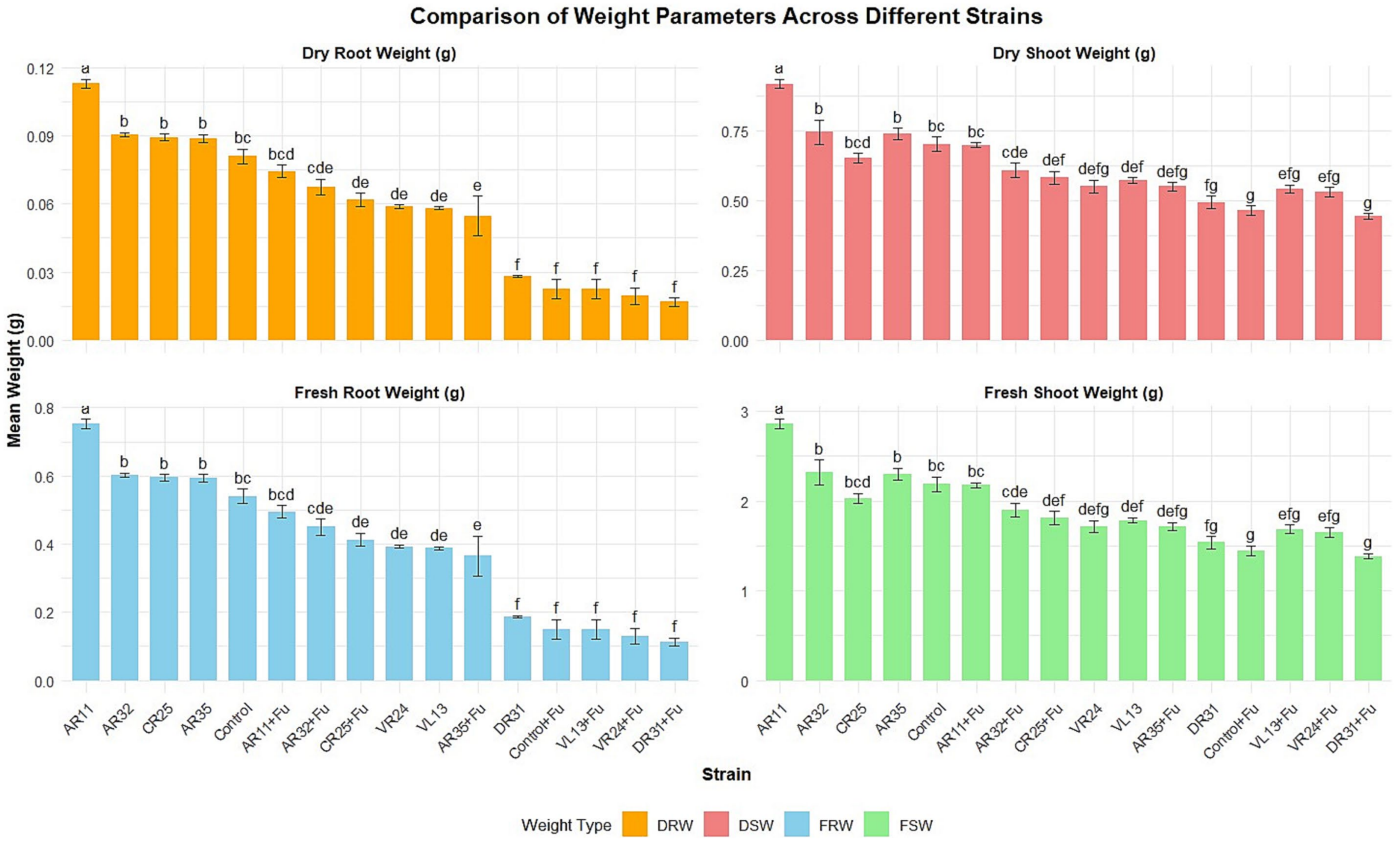
Effect of endophytic bacterial inoculation on pea root and shoot biomass (fresh and dry weight) under both non-stress and *Fusarium*-stressed conditions. Bars show mean ± SE (*n* = 4). Treatments include individual strains, co-inoculations with *Fusarium* (+Fu), and uninoculated controls. Different lowercase letters above bars indicate significant differences among means (*p* < 0.05).

Biomass data reinforced the observed trends in plant height (Figure 2). Under non-stress conditions, AR11 led to the highest shoot (2.88 ± 0.3 g fresh, 0.92 ± 0.05 g dry) and root (0.75 ± 0.2 g fresh, 0.11 ± 0.04 g dry) biomass, with all values falling within the top statistical group (“a”). In contrast, AR32 and AR35 resulted in significantly lower values (e.g., AR32: 2.37 ± 0.3 g shoot fresh, group “b”; 0.76 ± 0.05 g dry, group “b”). Under *Fusarium* challenge, AR11 maintained robust biomass accumulation, with shoot fresh and dry weights of 2.46 ± 0.2 g (group “d”) and 0.78 ± 0.03 g (group “d”), and root fresh and dry weights of 0.49 ± 0.2 g and 0.09 ± 0.02 g (group “d”), respectively. These values were significantly higher than those observed in the pathogen-only control (shoot dry weight: 0.49 ± 0.02 g; group “f”). Compared to AR11, AR32 and AR35 retained moderate biomass under stress but were statistically inferior. AR32-treated plants under infection conditions recorded 0.55 ± 0.03 g (shoot dry, group “f”) and 0.065 ± 0.03 g (root dry, group “e”), whereas AR35 showed even lower values (shoot: 0.47 ± 0.02 g, group “g”; root: 0.056 ± 0.02 g, group “f”). These distinctions confirm that AR11 consistently sustained significantly higher biomass accumulation under pathogen pressure.

### Effect of endophytic bacteria on chlorophyll content (SPAD value) under *Fusarium* challenge

**Figure 3:**
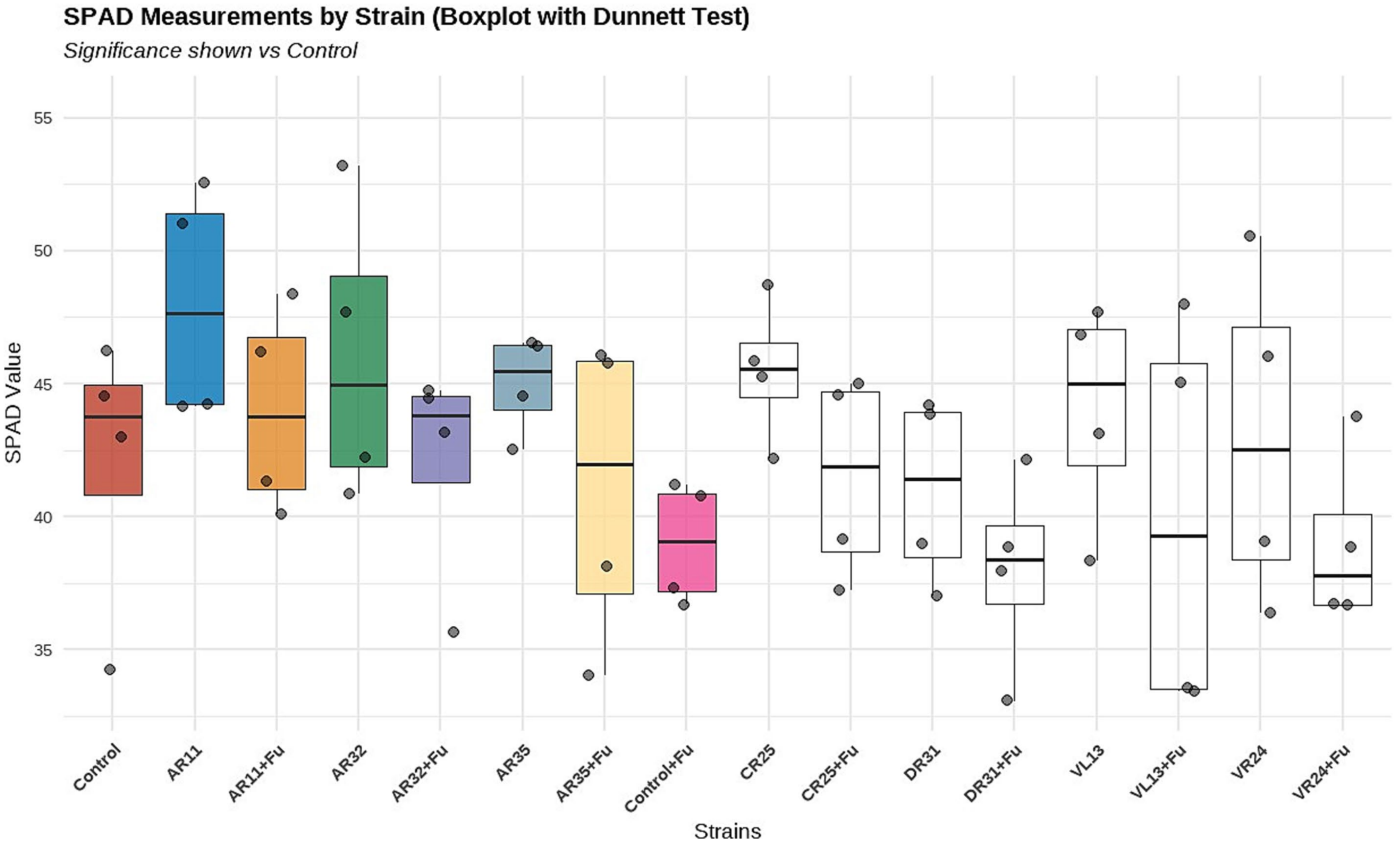
Effect of endophytic bacterial inoculation on chlorophyll content (SPAD index) in pea plants under non-stress (green boxplots) and *Fusarium*-stressed (yellow boxplots) conditions. Boxplots display the distribution of SPAD values across treatments, with individual data points representing biological replicates (*n* = 4).

Chlorophyll content, measured by SPAD index, significantly varied among treatments under both normal and *Fusarium*-infected conditions (Figure 3). In the absence of the pathogen, inoculation with strains AR11, AR32, and CR25 resulted in significantly higher SPAD values compared to the non-inoculated control (*p* < 0.05), indicating improved chlorophyll accumulation. Among these, AR11 exhibited the highest median SPAD value, followed by AR32 and CR25, as shown in the boxplot. Under *Fusarium* stress, the treatments AR11 + *Fusarium* and AR32 + *Fusarium* maintained significantly higher SPAD values compared to the *Fusarium*-only control, demonstrating their ability to alleviate pathogen-induced chlorophyll reduction. These statistical differences are supported by the Dunnett test results shown in Figure 3. In contrast, treatments such as VL13 + *Fusarium* and DR31 + *Fusarium* did not significantly differ from the *Fusarium* control, suggesting limited effectiveness under pathogen pressure. Overall, the data highlight the superior performance of AR11 and AR32 in supporting chlorophyll retention both in the presence and absence of *Fusarium* infection.

### Correlation between SPAD measurements and root/shoot development

**Figure 4:**
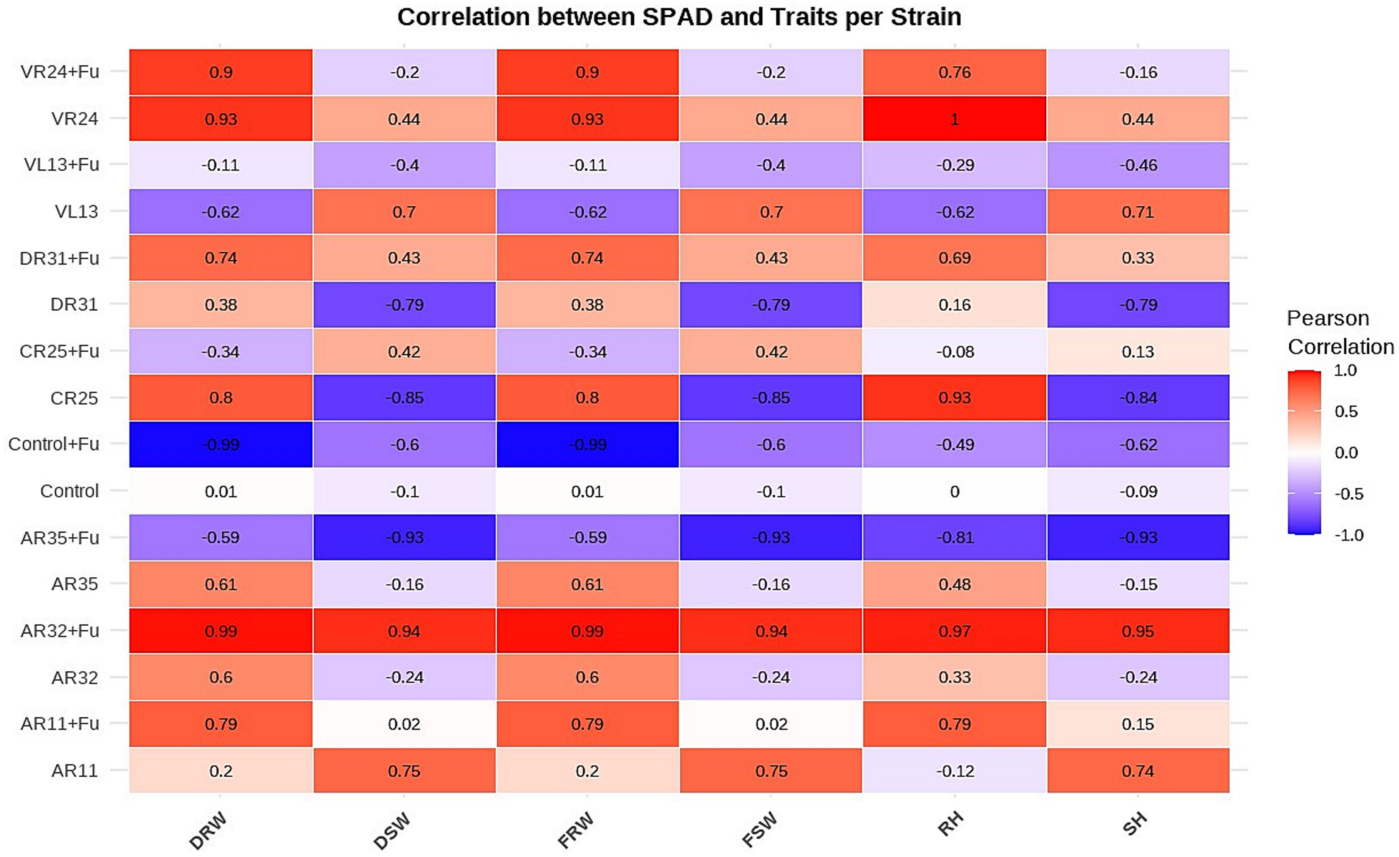
Correlation of SPAD index with plant growth parameters across bacterial treatments. The heatmap displays Pearson correlation coefficients between SPAD values (chlorophyll content) and plant growth traits dried root weight (DRW), dried shoot weight (DSW), fresh root weight (FRW), fresh shoot weight (FSW), root height (RH), and shoot height (SH), for each bacterial strain under both *Fusarium*-infected (+Fu) and non-infected conditions. Color intensity represents the strength and direction of correlation, with red indicating strong positive and blue indicating strong negative correlations.

Correlation analysis between SPAD index and plant growth traits revealed notable variation across bacterial treatments, with several strains showing strong associations between chlorophyll content and overall plant performance (Figure 4). AR32 + *Fusarium* exhibited the strongest and most consistent positive correlations across all measured traits, including fresh root weight (*r* = 0.99), dried shoot weight (*r* = 0.94), root height (*r* = 0.97), and shoot height (*r* = 0.95), indicating that SPAD values reliably reflected plant vigor under stress. AR11 + *Fusarium* also demonstrated strong positive correlations, particularly with fresh root weight (*r* = 0.79) and root height (*r* = 0.79), suggesting that AR11 not only preserved chlorophyll levels but also supported below-ground biomass development during *Fusarium* challenge. In the non-stressed condition, AR11 showed high correlations between SPAD and dried shoot weight (*r* = 0.75) and shoot height (*r* = 0.74), confirming its broader role in enhancing shoot growth. Additionally, VR24 + *Fusarium* displayed strong correlations between SPAD and most traits, including shoot weight and root-related parameters. In contrast, treatments such as Control + *Fusarium* and CR25 showed weak or negative correlations, indicating a disrupted or inconsistent relationship between chlorophyll content and growth under stress. These findings reinforce the role of AR32 and AR11 in maintaining a strong physiological link between photosynthetic capacity and biomass production under both normal and *Fusarium*-infected conditions.

### Disease severity

**Figure 5:**
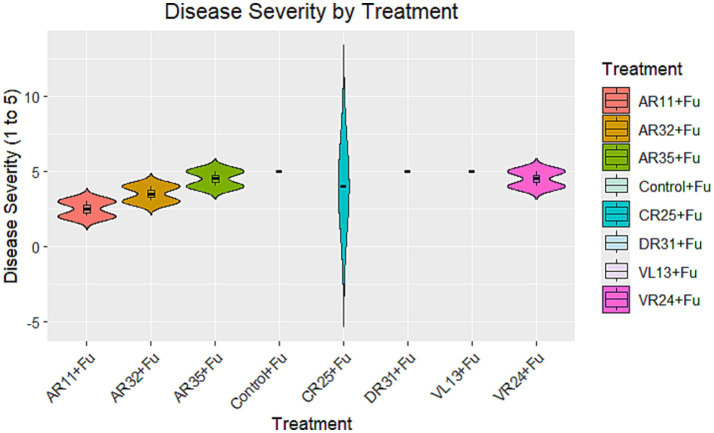
Disease severity by treatment group: violin plot showing the distribution of disease severity scores (y-axis is ranging from 1 to 5) for each treatment group. The boxplots within the violins represent the median and interquartile range.

The impact of different bacterial treatments on *Fusarium* disease severity varied notably. Treatments with AR11 + Fu, AR32 + Fu, and AR35 + Fu resulted in lower disease severity scores compared to the Control + Fu, indicating moderate to strong biocontrol activity (Figure 5). In contrast, the CR25 + Fu treatment showed disease severity levels like or higher than the Control + Fu, indicating it did not reduce disease symptoms. Likewise, DR31 + Fu, VL13 + Fu, and VR24 + Fu did not show significant effect on disease severity.

### Genome features of strain AR11

The whole genome sequencing strain AR11 using the PacBio SMRT platform produced a complete circular genome of 5.49 Mb (Table 1). Genome assembly and annotation predicted 5,175 coding DNA sequences (CDSs), along with 84 tRNA genes, seven copies each of the 16S and 23S rRNA genes, and 25 small RNAs (sRNAs). Additional structural features included 15 genomic islands, six prophage regions, three CRISPR loci, and six biosynthetic gene clusters (BGCs) related to secondary metabolism. Epigenomic profiling identified 61,116 N6-methyladenine (m6A) and 6,484 N4-methylcytosine (m4C) modifications, along with 251,055 unclassified modified bases.

**Table 1:** Summary of key genomic features identified in the AR11 genome *Serratia* sp.

Feature	Number/Value
Coding sequences (CDSs)	5,175
rRNA genes (16S, 23S)	7 copies each
tRNA genes	84
Small RNAs (sRNAs)	25
Genomic islands	15 (265.8 kb total)
Prophage regions	6 (409 kb total)
CRISPR loci	3
Secondary metabolite gene clusters	6 (25–49 kb)
m6A/m4C modified bases	61,116/6,484
Unclassified modified bases	251,055

Functional annotation of the genome revealed extensive database coverage. A total of 5,023 protein-coding genes (97.1%) received at least one functional annotation (Table 2). Annotation breakdown across major databases is summarized below. The majority of annotated genes were ≥300 bp in length, indicating well-supported predictions.

**Table 2:** Functional annotation coverage of *Serratia* sp. AR11 coding genes across reference databases.

Database	Total annotated genes	100–300 bp	≥300 bp
NR	5,022	2,263	2,329
TrEMBL	5,014	2,259	2,329
eggNOG	4,752	2,153	2,304
Pfam	4,636	2,078	2,275
GO	3,997	1,733	2,069
Swiss-Prot	3,755	1,583	1,988
KEGG	3,165	1,325	1,689
All annotated	5,023	2,263	2,329

**Figure 6:**
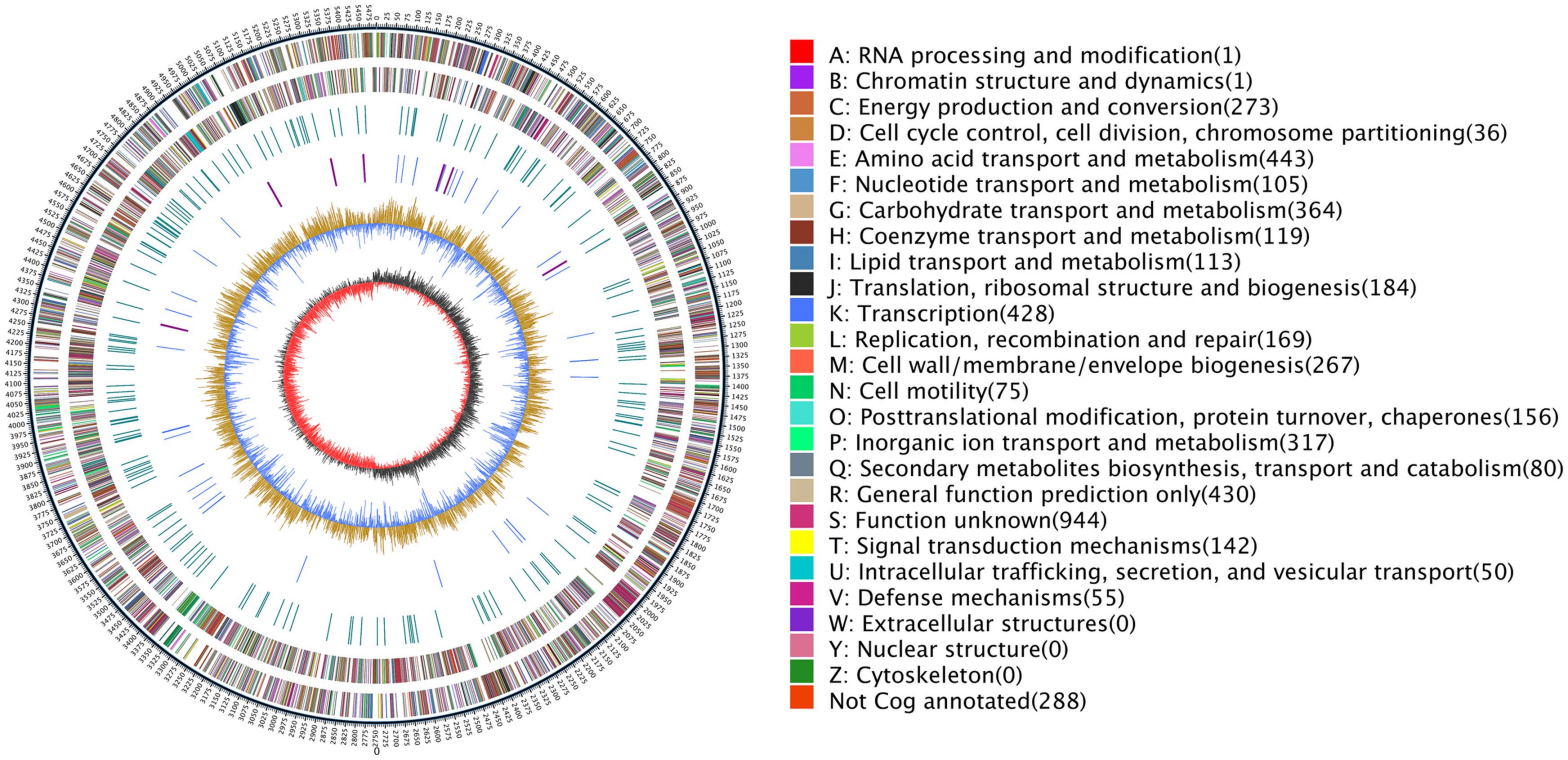
Circular genome map of strain AR11. The map displays genome coordinates, coding sequences (CDSs) color-coded by COG functional categories, and key categories such as amino acid transport, transcription, and carbohydrate metabolism. The innermost graph illustrates GC content and GC skew. Genes are color-coded based on their COG functional categories, with a comprehensive key detailing each category and its associated color provided immediately alongside the map.

Additional structural and regulatory features were identified across the AR11 genome. Repeat sequence analysis showed that approximately 0.14% of the genome consisted of interspersed and tandem repeats. DNA methylation profiling detected 61,116 N6-methyladenine (m6A) and 6,484 N4-methylcytosine (m4C) modifications, along with 251,055 unclassified modified bases; notably, GATC motifs were nearly fully methylated. Mobile genetic elements included 15 genomic islands (totaling 265.8 kb), six prophage regions (409 kb), and three CRISPR loci. Six secondary metabolite biosynthetic gene clusters ranging from 25 to 49 kb were also identified. The circular genome map of *Serratia* sp. AR11 (Figure 6) illustrates the genomic architecture, including coding sequence distribution, GC content and skew, and the functional categories of genes based on COG classification. The complete genome sequence has been deposited in the NCBI database under Bio Sample accession number SAMN49938280 (Submission ID: SUB15457842).

### Taxonomic affiliation based on NR annotation

**Figure 7:**
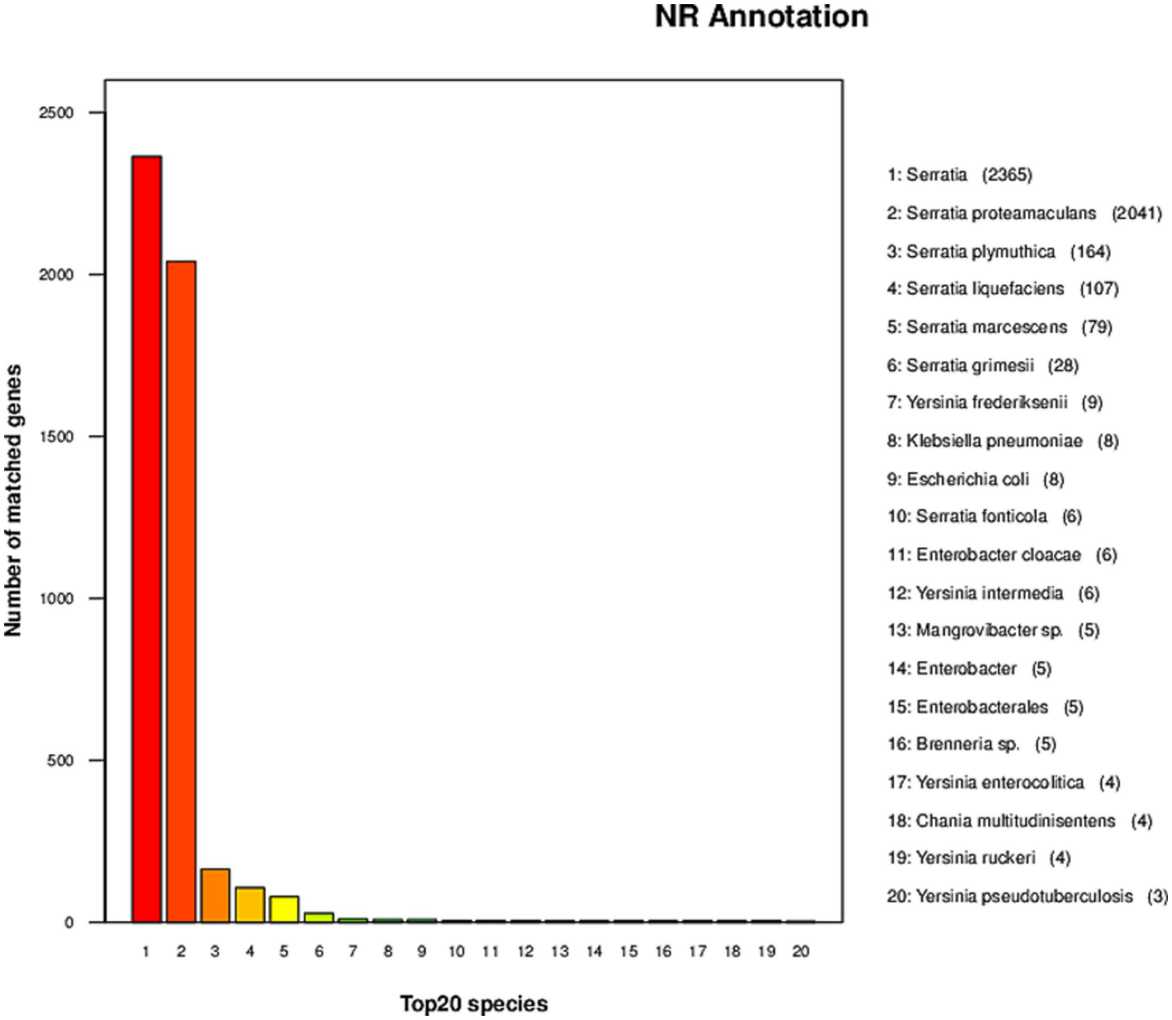
Taxonomic distribution of top NR matches. The bar plot depicts the number of predicted genes in the AR11 genome that matched protein sequences from the top 20 reference species in the NCBI NR database.

**Figure 8:**
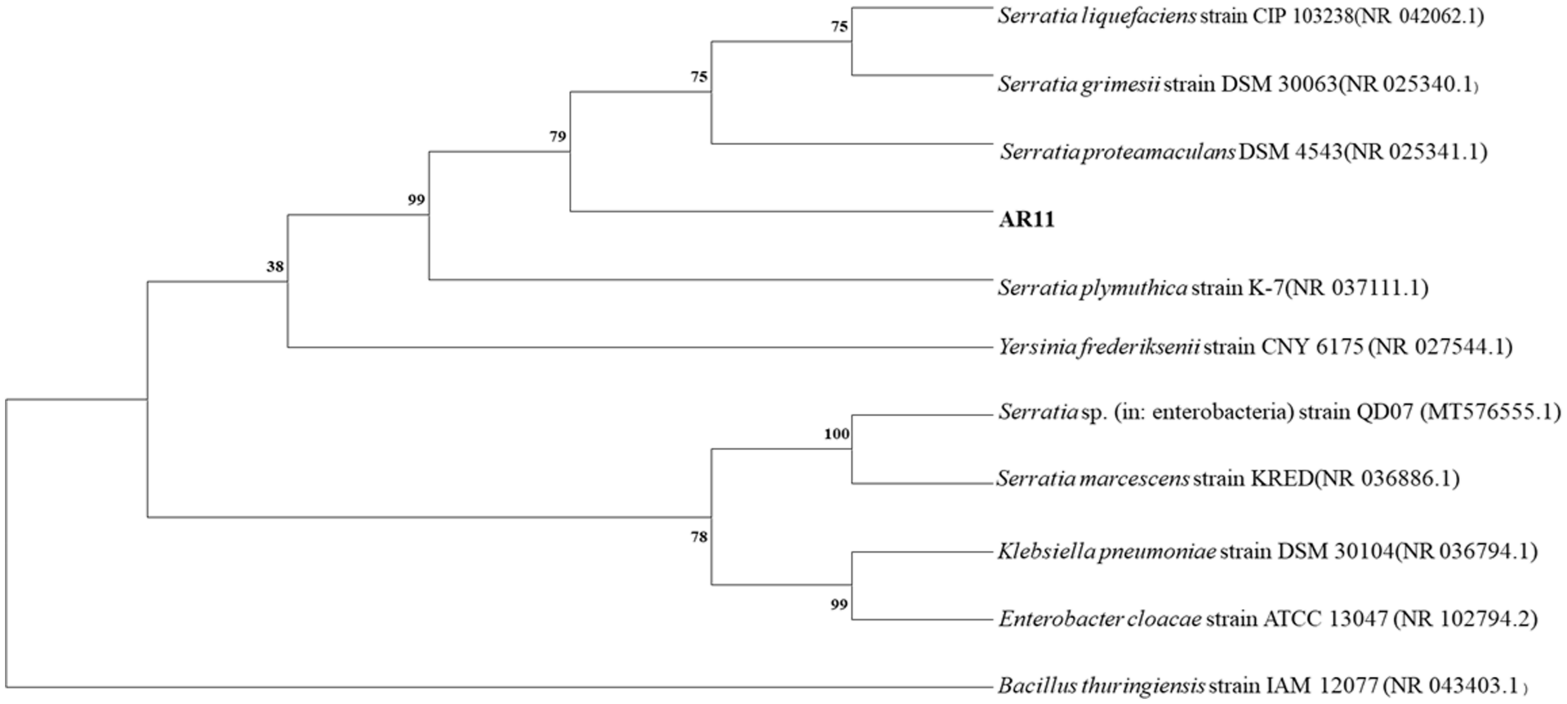
Neighbor-joining phylogenetic tree illustrating the evolutionary relationships of bacterial strains based on 16S rRNA gene sequences. Bootstrap support values (1,000 replicates) are shown at the nodes. Evolutionary distances were computed using the maximum composite likelihood method. The analysis included 11 nucleotide sequences, with a final dataset of 1,567 positions (pairwise deletion). All analyses were conducted in MEGA11. The position of isolate AR11 is indicated.

Strain AR11 was initially selected as *Bacillus* based on 16S rRNA gene analysis. However, whole-genome annotation against the NCBI non-redundant (NR) protein database revealed a different taxonomic affiliation. The majority of predicted protein-coding genes showed highest similarity to sequences from the genus *Serratia*. Specifically, 2,365 genes matched unclassified *Serratia* strains, and 2,041 matched *Serratia proteamaculans*. Additional matches were observed for *S. plymuthica* (164 genes), *S. liquefaciens* (107), *S. marcescens* (79), *S. grimesii* (28), and *S. fonticola* (6). In contrast, only a few gene matches were found with other genera, such as *Yersinia frederiksenii* (9 genes), *Klebsiella pneumoniae* (8), and *Escherichia coli* (8), further supporting the affiliation of AR11 with the genus *Serratia* (Figure 7). Due to multiple heterogeneous 16S rRNA gene copies in the genome, we selected the most abundant and representative sequence, based on coverage and similarity to *Serratia*, to construct the phylogenetic tree. This tree placed AR11 within the *Serratia* clade, closely related to *S. proteamaculans* (Figure 8). Average Nucleotide Identity (ANI) analysis using OrthoANIu (Yoon et al., 2017) further confirmed this placement, showing 95.69% identity between AR11 and *S. proteamaculans*, consistent with the species-level classification. In contrast, ANI with a representative *Bacillus* genome was only 63.35%, with genome coverage below 1%. These findings confirm classification of AR11 as *Serratia proteamaculans*, primarily supported by NR-based genome annotation, and demonstrate the limitations of relying solely on 16S rRNA for taxonomic assignment.

### Functional annotation and KEGG pathway classification

**Figure 9:**
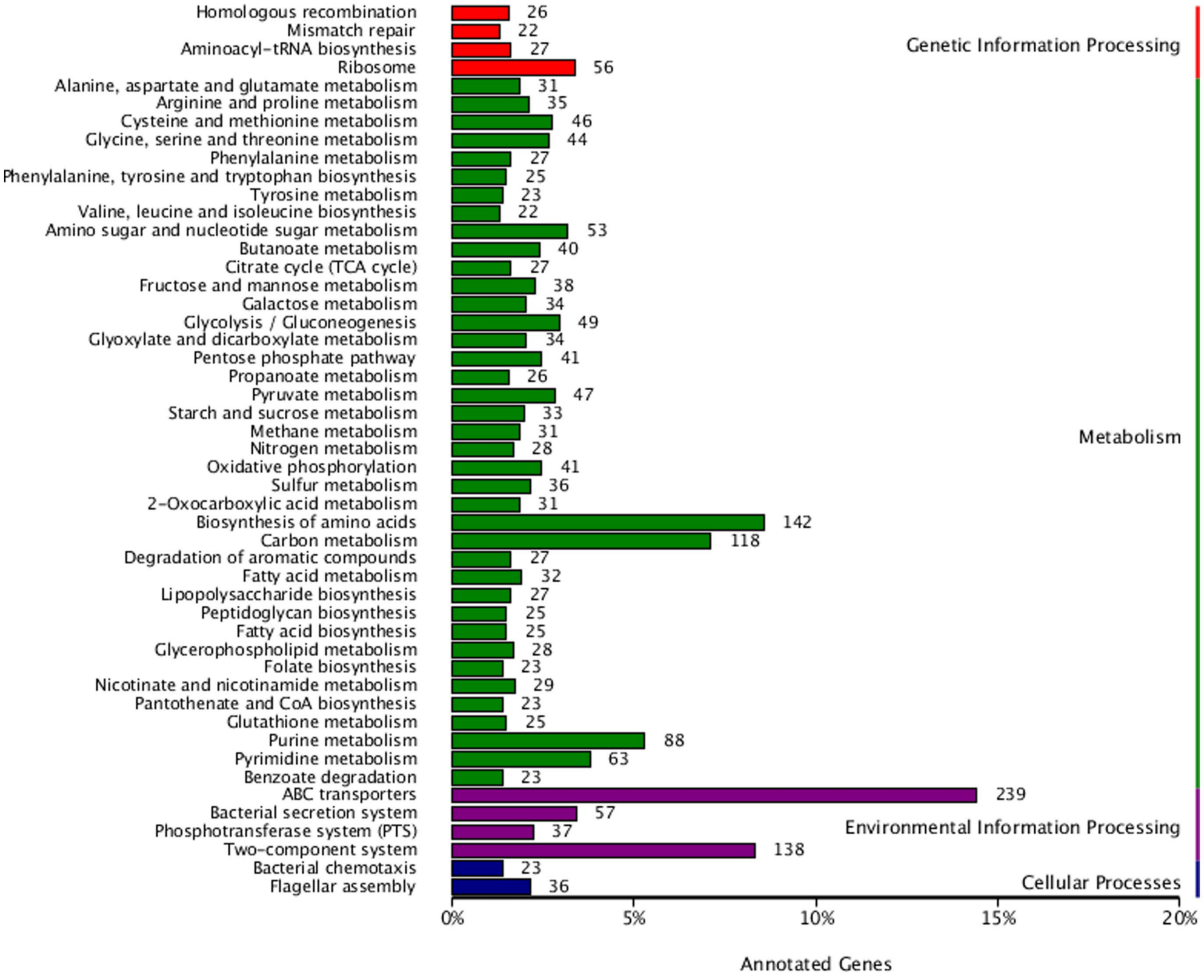
The KEGG pathway classification of AR11’s genome reveals high functional diversity, with major representation in amino acid and carbohydrate metabolism, energy production, ABC transporters, and two-component systems, emphasizing the strain’s metabolic adaptability and ecological competence.

KEGG pathway annotation of strain AR11 revealed a wide range of functional capabilities, highlighting its metabolic versatility and ecological adaptability. Among the annotated genes, 142 were associated with amino acid metabolism, 118 with carbohydrate metabolism, and 88 with energy metabolism (Figure 9). These dominant categories indicate strong potential for essential metabolic activities required for growth and survival. The genome also contained genes directly linked to plant growth-promoting traits. Specifically, 28 genes were involved in nitrogen metabolism, 37 in the phosphotransferase system (PTS), and 41 in oxidative phosphorylation indicating pathways critical for nutrient acquisition and energy production.

Strain AR11 has an enhanced ability to detect and respond to environmental changes. It contains 239 genes encoding ATP-binding cassette (ABC) transporters and 138 genes associated with two-component regulatory systems, which play key roles in environmental signal detection and cellular response. Additionally, genes related to bacterial chemotaxis (23), flagellar assembly (36), and multiple secretion systems were detected. Several genes associated with secondary metabolite biosynthesis and membrane transport were also identified, pointing toward potential antimicrobial activity and adaptation to plant-associated niches.

Screening of the AR11 genome against the Comprehensive Antibiotic Resistance Database (CARD) identified several genes associated with intrinsic resistance, including efflux pumps (adeF, msbA, emrR) and regulators (CRP), as well as sequence variants of elongation factor Tu and penicillin-binding protein PBP3.

### Functional annotation based on eggNOG classification

The genome of strain AR11 was annotated using the eggNOG database, which assigned its predicted proteins to 25 functional categories based on the Clusters of Orthologous Groups (COG) system. The most common category was R: General function prediction only, which included 944 proteins (19.37%), indicating a high number of proteins with unknown or putative functions.

**Figure 10:**
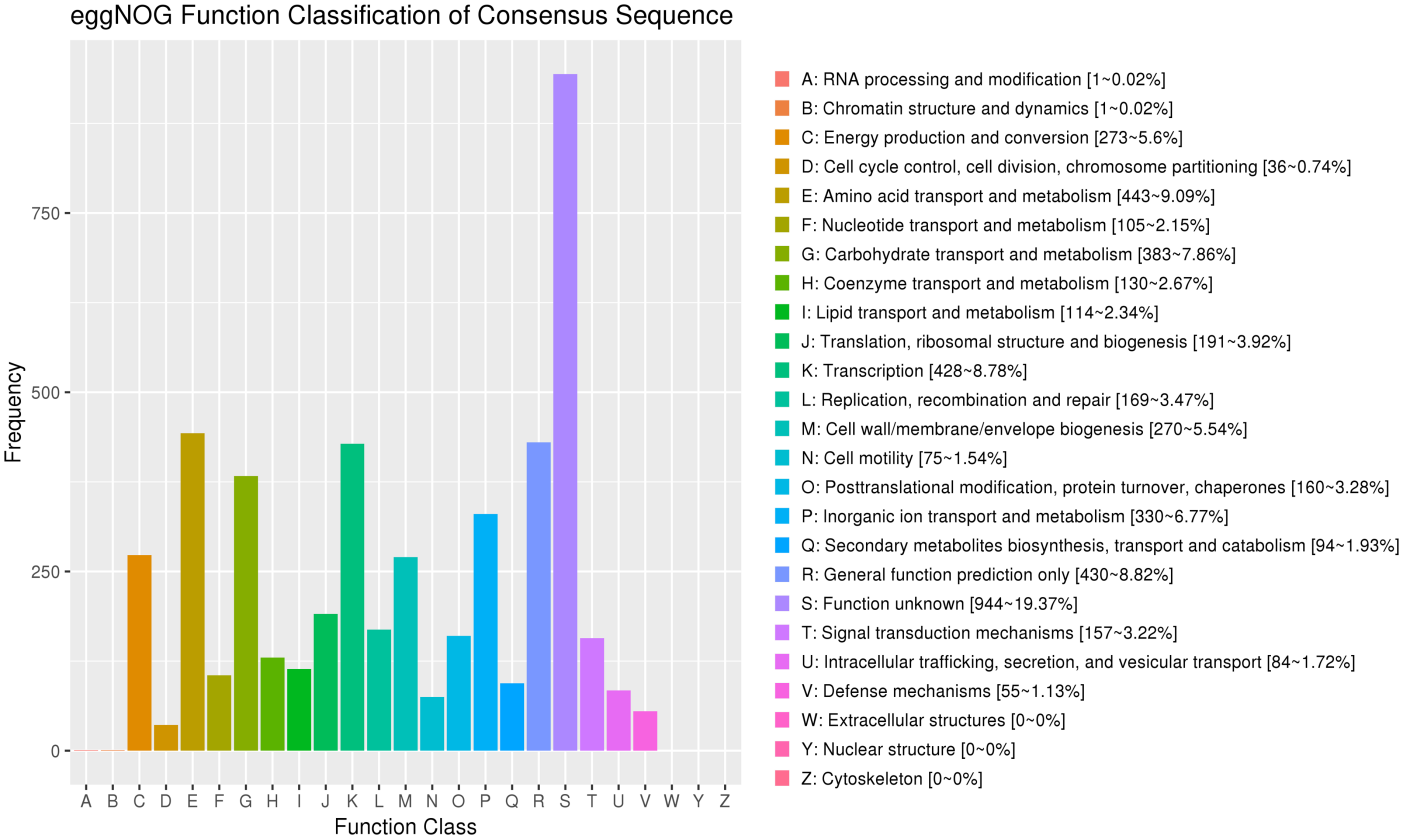
EggNOG functional classification of predicted proteins. The bar chart displays the distribution of predicted proteins across 25 COG categories. The most abundant category is R: general function prediction only, followed by categories associated with metabolism, transcription, and nutrient transport. The y-axis represents the number of proteins in each category. Protein classification highlights metabolic and regulatory diversity.

A large number of proteins were related to amino acid transport and metabolism (443 proteins, 9.09%), transcription (428 proteins, 8.78%), and carbohydrate transport and metabolism (383 proteins, 7.86%). These results suggest that strain AR11 has flexible metabolic pathways and effective systems for regulating gene expression. Among the functional categories, P: Inorganic ion transport and metabolism (330 proteins, 6.77%), C: Energy production and conversion (273 proteins), and H: Coenzyme transport and metabolism (142 proteins) were prominently represented. Additionally, proteins related to V: Defense mechanisms (55 proteins), U: Intracellular trafficking and secretion (84 proteins), and N: Cell motility (75 proteins) were identified, indicating potential roles in environmental interactions and motility (Figure 10). As expected for a prokaryotic organism, no proteins were assigned to categories W (extracellular structures), Y (nuclear structure), or Z (cytoskeleton).

### Gene Ontology enrichment analysis

**Figure 11:**
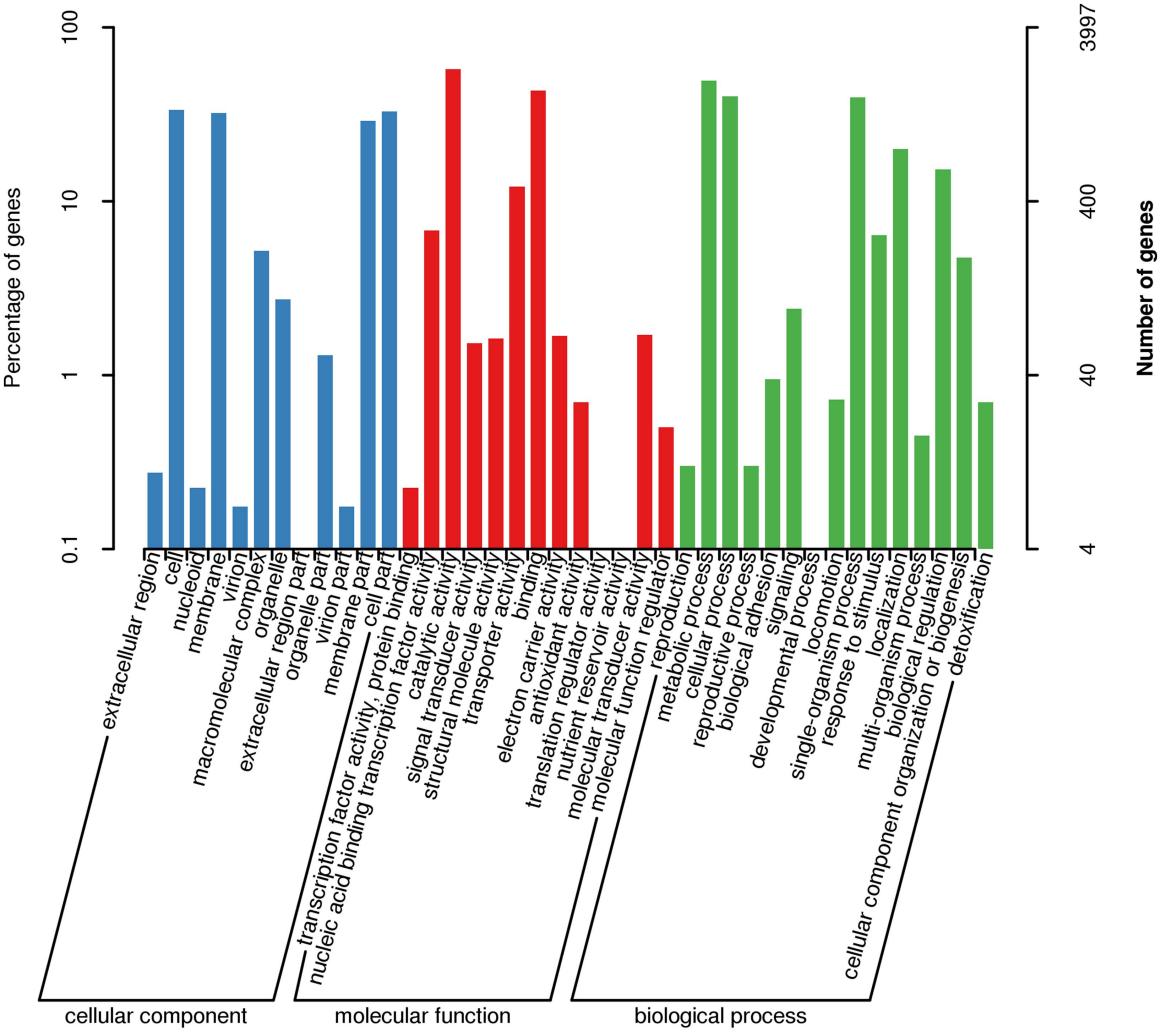
Gene Ontology (GO) enrichment analysis of the AR11 genome. Bar plots represent the number of genes associated with enriched GO terms across the three domains: biological process, molecular function, and cellular component.

Gene Ontology (GO) enrichment analysis of the AR11 genome categorized coding genes into three principal GO domains: biological process, molecular function, and cellular component (Figure 11). In the biological process category, approximately 700 genes were associated with metabolic processes, 650 with cellular processes, and 550 with single-organism processes. Additionally, around 400 genes were linked to transport, 300 to the regulation of biological processes, and 250 to the response to stimulus. Within the Molecular Function domain, catalytic activity was the most represented category (~600 genes), followed by protein binding (~500), nucleic acid binding (~400), and transporter activity (~350). In the cellular component category, genes were predominantly assigned to the cell (~450), membrane (~300), and membrane part (~200), indicating a notable prevalence of membrane-associated systems.

### Distribution of carbohydrate-active enzyme families

**Figure 12:**
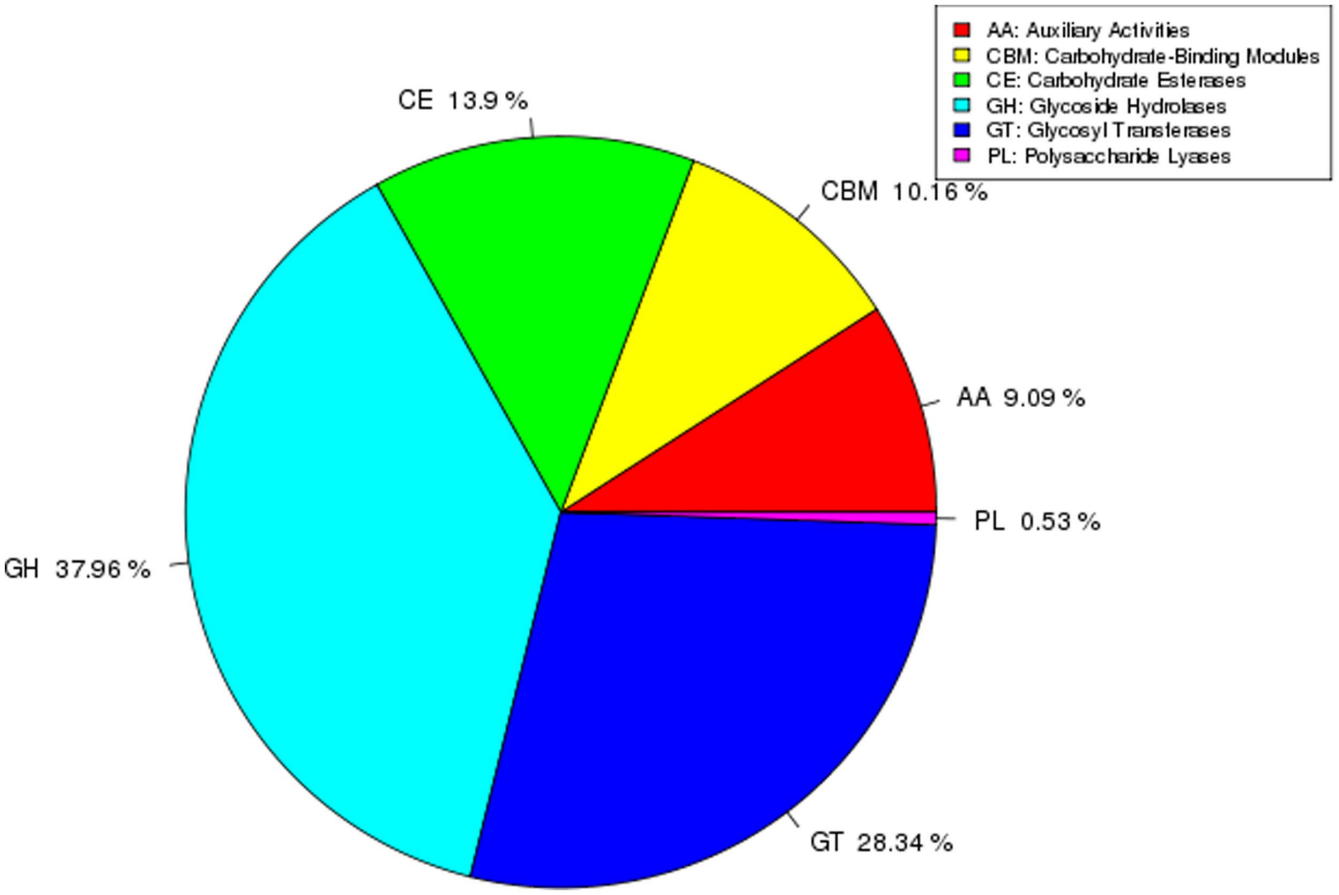
Distribution of CAZyme families in the AR11 genome. The pie chart illustrates the relative proportions of each CAZyme family identified in the genome. GHs and GTs were the most abundant, reflecting AR11’s capacity for complex carbohydrate metabolism and potential involvement in plant-microbe interactions.

The genome of strain AR11 encodes a diverse array of carbohydrate-active enzymes (CAZymes), classified into six major CAZy families (Figure 12). The glycoside hydrolases (GHs) family was the most predominant, comprising 37.96% of all CAZyme-encoding genes. These enzymes play a key role in the hydrolysis of complex polysaccharides into simpler sugars. The second most abundant family was glycosyltransferases (GTs), accounting for 28.34% of CAZyme genes, followed by carbohydrate esterases (CEs) at 13.9%, and carbohydrate-binding modules (CBMs) at 10.16%. Less abundant families included auxiliary activities (AAs), which make up 9.09% and are involved in oxidative reactions, and polysaccharide lyases (PLs), representing 0.53%, which cleave acidic polysaccharides through non-hydrolytic mechanisms.

### CRISPR-Cas system identification

To assess the genomic defense potential and adaptive immunity of *Serratia* strain AR11, an analysis of the CRISPR-Cas system was performed. Three distinct CRISPR loci were identified on Contig00001, each containing varying numbers of repeats and spacers (Table 3). The repeat sequences ranged from 22 to 30 base pairs, while the spacer sequences ranged from 24 to 31 base pairs. CRISPR.1 consisted of three repeats and two spacers, CRISPR.2 contained five repeats and four spacers, and CRISPR.3 included four repeats and three spacers. The presence of multiple CRISPR loci suggests the potential for a functional CRISPR-Cas system, which could play a role in the strain’s ability to defend against foreign genetic elements.

**Table 3:** CRISPR loci identification in *Serratia* strain AR11 genome.

CRISPR ID	Contig ID	Start	End	Repeat No.	Space No.
CRISPR.1	Contig00001	460,288	460,425	3	2
CRISPR.2	Contig00001	596,173	596,379	5	4
CRISPR.3	Contig00001	4,283,598	4,283,782	4	3

## Discussion

This study highlights *Serratia* sp. AR11 as a promising plant growth-promoting endophyte (PGPE) with dual biostimulant and biocontrol activity. Isolated from *Artemisia absinthium*, AR11 significantly enhanced pea plant growth and mitigated the deleterious effects of *Fusarium* infection. Through integrated phenotypic assays and high-throughput genomic analysis, it is indicated the molecular mechanisms are likely to underly these beneficial traits. These results are consistent with numerous studies reporting that endophytic and rhizosphere-associated *Serratia* species contribute to plant growth promotion and stress mitigation through diverse functional mechanisms (Kshetri et al., 2019; Kulkova et al., 2024; Patel et al., 2024).

### Genomic foundations of biostimulant activity

Under both normal and *Fusarium*-challenged conditions, *Serratia* sp. AR11 significantly enhanced root and shoot development in pea plants. This suggests that AR11 improves nutrient acquisition and enhances stress tolerance through its interaction with the root system. These beneficial effects are supported by the genomic enrichment of genes related to primary metabolism, energy generation, and nutrient transport. Functional annotations based on KEGG pathways and COG categories revealed a substantial number of genes involved in amino acid metabolism (443), carbohydrate metabolism (383), and inorganic ion transport and metabolism (330) traits commonly found in nutrient cycling rhizobacteria. Notably, the presence of 28 genes associated with nitrogen metabolism and 37 genes encoding components of the phosphotransferase system (PTS) indicates that AR11 can facilitate nitrogen assimilation and sugar uptake, both critical for plant growth and vigor. Similar metabolic profiles have been reported in well-characterized PGPRs such as *Streptomyces*, *Bacillus velezensis*, *Bacillus*, and *Pseudomonas fluorescens*, which are known to improve biomass accumulation and nutrient-use efficiency in host plants (Olanrewaju et al., 2021; Sanow et al., 2023; Xu et al., 2025). Moreover, the identification of 41 genes related to oxidative phosphorylation and 273 genes involved in energy production and conversion (COG category C) highlights AR11’s strong metabolic activity. This energy generating capacity likely supports its persistence and activity in both the rhizosphere and endospheric. Comparable energy-related gene profiles have been observed in PGPRs adapted to oligotrophic or nutrient-poor soils (Colin et al., 2017), supporting AR11’s suitability for use in low-input agricultural systems. Although genes specifically annotated phytohormone biosynthesis were not identified, the presence of key genes in the tryptophan biosynthesis pathway suggests that *Serratia* sp. AR11 may possess the capacity for auxin (IAA) production, a well-known plant growth-promoting trait in PGPRs. This genomic indication aligns with previous reports of IAA synthesis in *Serrati*a spp., particularly *S. plymuthica*, has been stimulate root elongation and enhance plant growth (Rico-Jiménez et al., 2023). Future studies involving hormone quantification assays could help validate the functional expression of these genomic traits *in planta*. This mechanism may also contribute to the improved root development observed in AR11-treated plants.

### Genomic drivers of biocontrol against *Fusarium*

The ability of AR11 to reduce stunting in *Fusarium*-infected pea plants appears to be supported by several genomic traits associated with biocontrol functions. Genome analysis revealed six distinct biosynthetic gene clusters (BGCs), each ranging from 25 to 49 kb in size, that are involved in the synthesis of secondary metabolites. Genome analysis identified six biosynthetic gene clusters potentially involved in secondary metabolite production, which may include compounds with antifungal properties contributing to *Fusarium* inhibition. While direct chemical profiling was beyond the scope of this study, these genomic features provide a valuable basis for future metabolomic validation to confirm the bioactive compounds and their roles in biocontrol. Similar antifungal compounds such as prodigiosin (Su et al., 2016), serratomolides (Marques-Pereira et al., 2020), and siderophores (Chlebek et al., 2022) have previously been reported in *Serratia* strains with demonstrated antifungal activity. Additionally, AR11’s genome contains a high number of glycoside hydrolases (GHs), as identified in the CAZyme analysis. These enzymes are known to break down fungal cell wall components, representing a direct antagonistic mechanism against fungal pathogens. Although direct chemical profiling was not conducted in this study, genome mining identified six biosynthetic gene clusters (BGCs) related to secondary metabolite production, including antifungal compounds. These *in silico* predictions, supported by CAZyme annotations, provide strong genomic evidence for the potential synthesis of bioactive molecules with antifungal activity. Such genomic insights offer a focused basis for future metabolomic validation. This finding is consistent with earlier studies on *Streptomyces* sp. GD-4, where GH-rich genomic regions contributed to antifungal activity and rhizosphere colonization (Xu et al., 2025). AR11 also appears to compete effectively with *Fusarium* and other soil microbes for space and nutrients. Its genome encodes 239 ABC transporters, 138 two-component regulatory systems, and numerous genes involved in amino acid and carbohydrate metabolism. ABC transporters help bacteria survive by protecting them from harmful compounds, helping them move toward plant roots, control gene activity, release useful substances, and take in nutrients (Zeng and Charkowski, 2021). These traits are essential for successful root colonization, exclusion of competing pathogens, and resource acquisition. Comparable features have been observed in *Bacillus subtilis* strains used for biocontrol in cucumber, where competitive interactions help limit pathogen establishment (Samaras et al., 2021). An additional key mechanism contributing to AR11’s biocontrol activity is induced systemic resistance (ISR), in which beneficial microbes prepare the plant’s immune system to respond more quickly and effectively to pathogen attack. AR11 contains 142 genes related to signal transduction (COG category T), along with 23 genes for chemotaxis and 36 for flagellar assembly, indicating its ability to sense plant signals and efficiently colonize root tissues. While induced systemic resistance (ISR) markers were not experimentally quantified, the genome of AR11 encodes key traits commonly associated with ISR-inducing microorganisms, including signal transduction systems, chemotaxis, and motility genes. These features indicate a strong potential to activate plant immune responses through ISR-related pathways. These traits are important because ISR is often triggered by microbial activity in the rhizosphere or within plant tissues. Previous studies have shown that ISR is a key defense mechanism induced by certain plant growth-promoting bacteria and fungi, which strengthens the plant’s resistance to a wide range of pathogens (Pieterse et al., 2014). Moreover, endophytic bacteria typically have more genes involved in motility and host communication such as those for chemotaxis, flagellar assembly, and pilus formation supporting their enhanced colonization and interaction with host plants (Jia et al., 2023). Similar ISR-inducing abilities have been reported in *Bacillus amyloliquefaciens*, which enhances plant defenses without directly attacking the pathogen (Salwan et al., 2023).

### Stress tolerance, genome plasticity, and epigenomic regulation

AR11 harbors several adaptive features for environmental resilience. The abundance of two-component systems and ABC transporters enables real-time sensing and response to rhizosphere changes (Borland et al., 2015). These systems are critical for PGPRs colonizing fluctuating environments such as sandy, degraded, or low-nutrient soils. Functional enrichment analyses of bacterial microbiota have shown that ABC transporters tend to be more abundant in rhizosphere communities compared to bulk soil, while two-component systems are often less represented. This shift may reflect the importance of ABC transporters in nutrient uptake and microbial competition in the rhizosphere, where microbes are exposed to intense biological and chemical interactions (Ruan et al., 2019). The high representation of both systems in AR11 suggests a robust ability to adapt to the dynamic conditions of the plant-root interface and maintain functional activity across diverse soil types. The genome of AR11 contains 15 genomic islands and six prophage regions, indicating past horizontal gene transfer events that may have contributed to its expanded metabolic functions and biocontrol capabilities. These regions often carry mobile genetic elements (MGEs), which are key drivers of bacterial evolution and adaptability. MGEs facilitate the acquisition and exchange of beneficial traits, such as stress tolerance, antimicrobial compound synthesis, and nutrient uptake. Their mobility allows bacteria to respond rapidly to environmental challenges, contributing to genetic diversity and ecological fitness (Weisberg and Chang, 2023). In beneficial microbes like AR11, this genomic flexibility enhances their ability to colonize the rhizosphere, interact with plant hosts, and adapt to changing soil conditions, all of which are critical for sustained plant growth promotion and pathogen suppression.

The CRISPR-Cas system serves as an adaptive immune mechanism in prokaryotes, enabling them to recognize and neutralize invading genetic elements such as bacteriophages and plasmids. This defense is achieved by incorporating short sequences from these invaders into the host genome, which are then used to guide the degradation of matching foreign DNA during subsequent attacks (Scrascia et al., 2023). In the genome of AR11, three CRISPR loci, with variable spacer-repeat architecture, were also identified. These provide a mechanism for defense against foreign DNA and enhance genome stability traits commonly reported in *Serratia* strains include Type I-E and I-F1 CRISPR systems (Scrascia et al., 2023). Epigenomic analysis of AR11 revealed widespread DNA methylation, particularly at GATC motifs targeted by Dam methyltransferases. This form of adenine methylation is a conserved epigenetic mechanism in bacteria, playing a crucial role in regulating gene expression, maintaining genomic stability, and facilitating adaptation to environmental stresses (Qiao et al., 2024). In *Serratia* and related enterobacteria, Dam-mediated methylation influences various cellular processes, including biofilm formation and stress responses, by modulating the transcription of specific genes (Bruneaux et al., 2022). The presence of these methylation patterns in AR11 suggests an additional layer of regulatory control that enhances its ability to thrive in diverse and challenging environments. The observed effects under controlled conditions highlight AR11 as a strong candidate for practical application. Given the genomic traits related to environmental adaptation, colonization, and functional versatility, field-level and semi-field trials are planned to evaluate performance under natural conditions, including interactions with native soil microbiota and environmental variability.

### Accurate taxonomic placement and safety considerations

The whole genome phylogenomic analysis reclassified strain AR11 as a member of the *Serratia proteamaculans* species, correcting its initial misidentification as *Bacillus* based on 16S rRNA sequencing. This reclassification is supported by Average Nucleotide Identity (ANI) values above the 95% species threshold and shared genomic characteristics with other *Serratia* strains known for plant growth-promoting and antifungal properties (Campos et al., 2024; Kulkova et al., 2024). To assess biosafety comprehensively, additional aspects such as horizontal gene transfer potential, environmental persistence, and possible non-target effects were considered. Biosafety evaluation of AR11’s genome revealed intrinsic antibiotic resistance genes related mainly to efflux pumps and regulatory proteins common in environmental bacteria. Importantly, no acquired antibiotic resistance or virulent genes associated with clinical pathogens were detected. Moreover, no conjugative elements, plasmids, or mobile resistance islands were identified, suggesting a low likelihood of horizontal gene transfer under field conditions. This supports AR11’s potential as a safe bioinoculant for sustainable agricultural use. While potential non-target effects on native symbionts such as rhizobia warrant future investigation, such interactions were not directly assessed in the current study. Consistent with previous recommendations, rigorous screening for pathogenicity and resistance genes is essential before field application of plant growth-promoting bacteria (Keswani et al., 2019). Given its genomic traits and observed performance, strain AR11 could be further developed for formulation as a bioinoculant or used as a model for selecting similar strains from other medicinal plants.

## Conclusion

This study presents *Serratia* sp. AR11, isolated from *Artemisia absinthium*, as a distinct endophytic strain with combined plant growth-promoting and disease-suppressing effects in pea under *Fusarium* stress. Its strong performance in controlled greenhouse conditions, including improved growth, biomass, chlorophyll content, and reduced disease severity, highlights its practical potential. Genome analysis supports these observations, revealing genes related to nutrient metabolism, stress response, and the predicted synthesis of antifungal compounds. While metabolite production was not experimentally confirmed, the genomic data provides a strong foundation for further functional validation. Unlike many previously reported *Serratia* strains, AR11 combines a unique plant origin with a clear genomic and phenotypic profile, making it a valuable candidate for developing bioinoculants tailored to pea cropping systems. The absence of high-risk virulence or resistance genes further supports its safe application. Follow-up studies focusing on metabolomic assays and field trials will help confirm and extend these findings. Future studies integrating metabolomic, microbiome, and formulation approaches may further enhance the application potential of strain AR11.

## Data Availability

The data presented in this study are available in the NCBI BioSample repository under accession numbers SAMN49938280 and PRJNA1290800.
